# Association of Infection With Human Papillomavirus and Development of End-Stage Kidney Disease in Taiwan

**DOI:** 10.1001/jamanetworkopen.2020.22107

**Published:** 2020-10-22

**Authors:** Renin Chang, Ming Li Chen, Cheng-Li Lin, Yao-Min Hung, James Cheng-Chung Wei

**Affiliations:** 1 Department of Emergency Medicine, Kaohsiung Veterans General Hospital, Kaohsiung, Taiwan; 2 Department of Recreation Sports Management, Tajen University, Pingtung, Taiwan; 3 Department of Medicine, Chung Shan Medical University Hospital, Taichung, Taiwan; 4 Management Office for Health Data, China Medical University Hospital, Taichung, Taiwan; 5 Department of Internal Medicine, Kaohsiung Municipal United Hospital, Kaohsiung, Taiwan; 6 Shu-Zen Junior College of Medicine and Management, Kaohsiung, Taiwan; 7 Tajen University, Pingtung, Taiwan; 8 Institute of Medicine, Chung Shan Medical University, Taichung, Taiwan; 9 Division of Allergy, Immunology and Rheumatology, Chung Shan Medical University Hospital, Taichung, Taiwan; 10 Graduate Institute of Integrated Medicine, China Medical University, Taichung, Taiwan

## Abstract

**Importance:**

The association between human papillomavirus (HPV) infection status and the natural process of kidney diseases has been neglected as an area of research. Further studies are needed to clarify factors that may alter the progression of end-stage kidney disease (ESKD).

**Objective:**

To describe the rates of ESKD among patients with and without HPV infection.

**Design, Setting, and Participants:**

In this nationwide, population-based retrospective cohort study, data were collected from the National Health Insurance Research Database of Taiwan. A total of 76 088 individuals with HPV infection were enrolled from January 1, 2000, to December 31, 2012, and compared with a control group of 76 088 individuals who had never been diagnosed with HPV infection (at a 1:1 ratio propensity-score matched by age, sex, index year, and comorbidities) in the context of the risk of developing ESKD. Statistical analysis was performed between November 2019 and July 2020.

**Exposures:**

HPV infection was defined according to the *International Classification of Diseases, Ninth Revision, Clinical Modification* codes*.*

**Main Outcomes and Measures:**

The main outcome was ESKD, as recorded in the Catastrophic Illness Patients database. Cox proportional hazards models were used to estimate hazard ratios (HRs) and 95% CIs, with the control group as a reference.

**Results:**

Of 152 176 individuals (79 652 [52.3%] women; mean [SD] age, 34.4 [19.1] years), 76 088 individuals (50.0%) had HPV and 463 individuals (0.3%) developed ESKD. Incidence of ESKD was lower in individuals with HPV history than in those without HPV history (3.64 per 10 000 person-years vs 4.80 per 10 000 person-years). In the fully adjusted multivariate Cox proportional hazards regression model, individuals with a history of HPV infection had a significant decrease in risk of ESKD (adjusted HR, 0.72; 95% CI, 0.60-0.87) after adjusting for demographic characteristics, comorbidities, and comedications. In the subgroup analysis, individuals ages 50 to 64 years with HPV infection had a statistically significantly lower risk of ESKD compared with individuals ages 50 to 64 years with no HPV infection (adjusted HR, 0.48; 95% CI, 0.34-0.68; *P* < .001), while there was no significant reduction in risk for the other age groups (ie, 0-19, 20-49, and 65-100 years).

**Conclusions and Relevance:**

In this study, a history of HPV infection was associated with a lower risk of subsequent ESKD. The mechanism behind this protective association remains uncertain. Future studies are required to clarify the possible biological mechanisms.

## Key Points

**Question:**

What is the association of human papillomavirus (HPV) infection with subsequent development of end-stage kidney disease (ESKD) in the general population of Taiwan?

**Findings:**

In this nationwide, population-based matched cohort study of 152 176 individuals, HPV infection was negatively associated with development of ESKD.

**Meaning:**

These findings suggest that future detailed studies of the mechanisms involved in the association between HPV infection status and decreased risk of ESKD could yield targets for interventions to delay the development of ESKD.

## Teaser

This cohort study investigates the association of human papillomavirus infection status with development of end-stage kidney disease in Taiwan.

## Introduction

End-stage kidney disease (ESKD) remains an important health issue globally.^1,2^ The disease burden of ESKD has increased,^4,5,6^ with a 2013 study^3^ finding more than 1.4 million people worldwide requiring kidney replacement therapy. As health care expenditures continue to increase, public health strategies that maintain costs and quality of care have focused on patients who are at high risk and who account for most health care costs.^7,8,9,10^ Taiwan has a high prevalence of kidney diseases. In a 2018 study,^11^ the estimated prevalence of chronic kidney disease (CKD) in Taiwan was 9.1% to 15.5% for CKD stages 3 to 5. Additionally, a previous study in a Taiwanese cohort revealed that the 8-year cumulative incidence in the ESKD rate among individuals with diabetic nephropathy was 14.5% (95% CI, 13.9-15.0) for people using traditional Chinese medicine and 16.6% (95% CI, 16.0-17.2) for people who did not use traditional Chinese medicine .^12^ A 2017 study^13^ found that in a Taiwanese population, a family history of ESKD was a risk factor associated with developing ESKD, suggesting a moderate level of heritability in the Taiwanese population. In addition to well-known risk factors of ESKD, such as diabetes, hypertension, dyslipidemia, and advanced age, infection-related kidney disease has become a subject of recent research.^3,14^ Studies have suggested that viral infections, such as hepatitis B virus (HBV), hepatitis C virus (HCV), HIV, dengue virus, and Hantaan virus, are associated with nephropathies, including vasculitis and immune complex proliferative glomerulonephritis.^14^ Several large cohorts have documented increased rates of CKD in the setting of HIV infection.^15,16^ Studies have also associated HCV infection with histopathological lesions in both native and transplanted kidneys, as well as in CKDs.^17,18^

Human papillomavirus (HPV), a double-stranded DNA virus, is known to be a distinct risk factor for multiple cancers. The oncogenesis mechanism related to this virus was elucidated in previous studies. This virus exclusively infects epithelium cells, interrupts the normal cell cycle, and triggers uncontrolled cell division.^19^ Although different HPV subtypes may infect different target cells and the persistence of viral genomes may be different, it is known that 2 HPV viral proteins (ie, E6 and E7) play crucial roles in the viral replication strategy for all subtypes.^20^ These viral proteins mediate oncogenesis progression by preventing apoptosis and causing cell-cycle arrest of the host cells.^21^

Owing to the occurrence of prolonged immunosuppression, HPV is a common infection in people who have received kidney transplants. Numerous studies have shown that HPV prevalence is significantly increased among individuals with established kidney transplants.^22^ Reactivation of oncogenic viruses, such as human herpes virus type 8, Epstein-Barr virus, HBV, HCV, and HPV, is also common among individuals with kidney transplants.^23^ Kidney transplants are also associated with ESKD, as the reason for kidney transplant is often to improve the quality of life of a person with ESKD.^24^ Owing to the long preinvasive state of precancer lesions, many HPV-related cancers are preventable with the help of screening and targeted treatment of HPV-related diseases. Thus, pretransplant vaccination and HPV screening in kidney transplant recipients are crucial.^25^ Because ESKD is frequently a cause of future kidney transplantation and HPV reactivation is an important issue in kidney transplantation, it is reasonable to examine the associations between history of HPV infection and subsequent development of ESKD. To further explore the association between HPV and ESKD, we analyzed a large longitudinal, nationwide cohort.

## Methods

This cohort study was approved by the institutional review board of the China Medical University Hospital in Taiwan. The need for informed consent was waived because the data were used anonymously and, in accordance with Taiwan’s Personal Information Protection Act,^26^ the identities of individuals with insurance were recoded before analysis. The study followed the Strengthening the Reporting of Observational Studies in Epidemiology (STROBE) reporting guideline.

### Data Source

This population-based retrospective cohort study used the Longitudinal Health Insurance Database (LHID) 2000, a medical claims database that includes 1 million randomly selected individuals insured from 1996 to 2000 by the National Health Insurance (NHI) system of Taiwan. The LHID was sampled from Taiwan’s National Health Insurance Research Database (NHIRD). In 1996, of 21.52 million residents of Taiwan, 20.04 million individuals (93%) were covered by NHI health care. By 2011, of 23.22 million residents of Taiwan, 23.20 million individuals (99%) were covered by NHI health care. The LHID gives the age and sex distributions of the general population and includes multiple claims files, such as those related to expenditure and order of ambulatory and inpatient care, the prescription of drugs, and the registry for beneficiaries. Together, these data provide information about the diagnoses and details of the use of health care. The diagnostic codes of the claims are recorded according to the *International Classification of Diseases, Ninth Revision, Clinical Modification (ICD-9-CM)*.^27^

### Study Design and Population

HPV infection was defined according to *ICD-9-CM* codes (ie, 079.4, 078.1, 078.10-078.12, 078.19, 795.05, 795.09, 795.15, 795.19, 796.75, and 796.79), and the inclusion criteria required at least 3 outpatient visits or any inpatient diagnosis. The HPV group consisted of 76 088 individuals ages 0 to 100 years and newly diagnosed with HPV infection from January 1, 2000, through December 31, 2012; individuals who were diagnosed with HPV from 1997 to 1999 were excluded. The date of the first HPV diagnosis was the index date, and this date was assigned to the matched unexposed individual with the same enrollment criteria. Thus, the study included 76 088 individuals who were newly diagnosed with HPV without an ESKD or kidney transplantation before the index date. These individuals, who were considered exposed, were compared with 76 088 individuals who were not exposed (non-HPV group) and who were matched, via propensity scores matching, by sex, age, index date, comorbidity status, and comedication.

The groups were matched by propensity scores through nearest-neighbor matching, initially to the eighth digit and then as required to the first digit. Therefore, matches were first made within a caliper width of 0.0000001, and then the caliper width was increased for unmatched cases to 0.1. We reconsidered the matching criteria and performed a rematch (using a greedy algorithm). For each individual in the HPV group, the corresponding comparisons were selected based on the nearest propensity score. The propensity scores were calculated using the probability of HPV assignment by using a logistic regression model and included the following baseline variables: year of index date, age, sex, comorbidities (ie, hypertension, diabetes, hyperlipidemia, coronary artery disease, cerebrovascular disease, CKD, chronic obstructive pulmonary disease [COPD], chronic liver diseases, systemic lupus erythematosus, ankylosing spondylitis, rheumatoid arthritis, Sjögren syndrome, HCV, HIV, and HBV), and medications (ie, statins, nonsteroidal anti-inflammatory drugs, and antihypertensive drugs). Individuals in the study group and control group were tracked from index visit until an ESKD event, withdrawal from the NHI program, or December 2013.

### Covariates and Outcome

The comorbidities analyzed in this study were hypertension (*ICD-9-CM* codes 401-405), diabetes (*ICD-9-CM* code 250), hyperlipidemia (*ICD-9-CM* code 272), coronary artery disease (*ICD-9-CM* codes 410-414), cerebrovascular disease (*ICD-9-CM* codes 430-438), CKD (*ICD-9-CM* codes 585.1-585.5), COPD (*ICD-9-CM* codes 490-492 and 493-496), chronic liver disease (*ICD-9-CM* codes 571.1, 571.2, 571.4, and 571.5), systemic lupus erythematosus (*ICD-9-CM* code 710.0), ankylosing spondylitis (*ICD-9-CM* code 720.0), rheumatoid arthritis (*ICD-9-CM* code 714.0), Sjögren syndrome (*ICD-9-CM* code 710.2), HCV infection (*ICD-9-CM* codes 070.44, 070.51, 070.54, 070.7, and V02.62), HIV infection (*ICD-9-CM* code 042), and HBV infection (*ICD-9-CM* codes 070.2, 070.3, and V02.61).

The outcome was patients with ESKD recorded in the Catastrophic Illness Patients (CIP) database who underwent maintenance dialysis therapy for at least 90 days. In the Taiwan health insurance system, individuals fitting the relevant category of catastrophic illness are classified as individuals who benefit from the catastrophic illness–associated treatment and do not need to pay the fee for that treatment. Verification of the eligibility for CIP certification requires strict investigation. For instance, a patient who has relevant diagnosis codes for ESKD and has received kidney replacement therapy for at least 3 months can apply for the certification.

### Statistical Analysis

The first ESKD diagnosis of each participant within the study period was used to calculate the risk of ESKD. Follow-up was conducted from the index date until withdrawal from the NHI program or until December 31, 2013. The incidence density of ESKD per 10 000 person-years was calculated in both groups. We used propensity score matching to control for sampling bias. Propensity scores represented patients’ probability of ESKD incidence, and the scores were determined by a multivariate logistic regression model. For balanced covariate distribution, the standardized difference was used to compare the mean of the binary variable between 2 groups in propensity score–matched and nonmatched samples. A standardized mean difference of less than 0.1 indicated a negligible difference in the distribution of a covariate between the HPV and non-HPV groups.

To investigate the independent association of HPV with ESKD, a Cox proportional hazard regression analysis was conducted to estimate the hazard ratios (HRs) and 95% CIs after adjusting for full covariates in the study. Variables found to be statistically significant in the univariable model were further examined in the multivariable model. To validate the robustness of the main study findings, a sensitivity analysis was performed by adjusting for demographic characteristics and comorbidities. The Kaplan-Meier curves were plotted to describe the cumulative incidence of ESKD in the 2 groups, and the long-rank test was performed to compare differences between the 2 groups. All statistical tests were 2-sided, and *P* values of .05 or less were considered statistically significant. All data and statistics were processed and analyzed by SAS statistical software version 9.3 (SAS Institute). The statistical analysis was performed between November 2019 and July 2020.

## Results

Of 152 176 participants (79 652 [52.3%] women) aged 0 to 100 years (mean [SD] age, 34.4 [19.1] years), 76 088 individuals (50%) had newly diagnosed HPV infection (39 738 women [52.2%]; mean [SD] age, 33.5 [18.8] years) and 463 individuals (0.3%) developed ESKD; 76 088 individuals who had no HPV infection (39 914 women [52.5%]; mean [SD] age, 33.7 [18.8] years) were matched by age, sex, and comorbidities (Table 1). Among 76 088 study participants with HPV infection, 60 877 individuals (80.0%) were age 49 years or younger. Propensity score matching resulted in 76 088 matched individuals in each group. The baseline characteristics were found to be well balanced between the groups after matching. The median (SD) follow-up time was 7.29 (3.61) years in the exposed group and 7.14 (3.62) years in the unexposed group. The group with HPV infection in this study, compared with the group without HPV infection, had similar proportions of statin use (3236 individuals [4.3%] vs 3161 individuals [4.2%]; standardized mean difference, 0.01) and antihypertensive drug use (12 757 individuals [16.8%] vs 12 672 individuals [16.7%]; standardized mean difference, <0.01) but a higher proportion of nonsteroidal anti-inflammatory drug (NSAID) use (47 525 individuals [62.5%] vs 42 194 individuals [55.5%]; standardized mean difference, 0.14).

**Table 1.** Baseline Patient Characteristics

Variables	Individuals, No. (%)		Standardized mean differences^c^
	Without HPV (n = 76 088)^a^	With HPV (n = 76 088)^b^	
Age, y			
<20	21 426 (28.2)	21 802 (28.7)	0.01
20-49	39 448 (51.9)	39 075 (51.4)	0.01
50-64	9484 (12.5)	9423 (12.4)	0.002
≥65	5730 (7.5)	5788 (7.6)	0.003
Mean (SD)	33.7 (18.8)	33.5 (18.8)	0.01
Sex			
Women	39 914 (52.5)	39 738 (52.2)	0.01
Men	36 174 (47.5)	36 350 (47.8)	0.01
Comorbidity			
Hypertension	10 194 (13.4)	10 285 (13.5)	0.004
Diabetes	1991 (2.62)	1918 (2.52)	0.01
Hyperlipidemia	8297 (10.9)	8643 (11.4)	0.01
Coronary artery disease	5476 (7.20)	5634 (7.40)	0.01
Cerebrovascular disease	854 (1.12)	834 (1.10)	0.003
Chronic kidney disease	545 (0.72)	526 (0.69)	0.003
COPD	5022 (6.60)	5169 (6.79)	0.01
Chronic liver diseases	6791 (8.93)	7120 (9.36)	0.02
Systemic lupus erythematosus	43 (0.06)	73 (0.10)	0.01
Ankylosing spondylitis	270 (0.35)	241 (0.32)	0.01
Rheumatoid arthritis	78 (0.10)	95 (0.12)	0.01
Sjögren syndrome	24 (0.03)	44 (0.06)	0.01
Hepatitis C	654 (0.86)	612 (0.80)	0.006
HIV	45 (0.06)	52 (0.07)	0.004
Hepatitis B	2289 (3.01)	2355 (3.10)	0.005
Comedications			
Statin	3161 (4.2)	3236 (4.3)	0.01
NSAID	42 194 (55.5)	47 525 (62.5)	0.14
Antihypertensive drug	12 672 (16.7)	12 757 (16.8)	0.003

Abbreviations: COPD, chronic obstructive pulmonary disease; HPV, human papillomavirus; NSAID, nonsteroidal anti-inflammatory drug.

^a^ Mean (SD) follow-up time, 7.14 (3.62) years.

^b^ Mean (SD) follow-up time, 7.29 (3.61) years.

^c^ A standardized mean difference of 0.1 or less indicates an insignificant difference.

In univariate Cox regression analysis and multivariate Cox regression analysis, individuals with HPV infection had a lower risk of developing ESKD compared with those without HPV infection, with an incidence rate of 3.64 per 10 000 person-years vs 4.80 per 10 000 person-years. (Table 2). The individuals who had histories of HPV infection were less likely to develop ESKD (unadjusted HR, 0.76; 95% CI, 0.63-0.91). Two kinds of multivariate Cox regression analysis showed a negative association of HPV infection with subsequent development of ESKD. After adjusting for demographic variables, comorbidities, and comedications at baseline, individuals with HPV infection had a 28% lower risk of developing ESKD vs those without HPV, with an adjusted HR of 0.72 (95% CI, 0.60-0.87). After adjusting for demographic characteristics and comorbidities, the adjusted HR was 0.71 (95% CI, 0.59-0.86).

**Table 2.** End-Stage Kidney Disease Incidence Rate and Risk Factors

Variable	Events, No.	Person-years	Rate^a^	HR (95% CI)		
				Crude	Model 1^b^	Model 2^c^
HPV						
No	261	543 584	4.80	1 [Reference]	1 [Reference]	1 [Reference]
Yes	202	554 369	3.64	0.76 (0.63-0.91)^d^	0.72 (0.60-0.87)^e^	0.71 (0.59-0.86)^e^
Age, y						
<20	4	332 501	0.12	1 [Reference]	1 [Reference]	1 [Reference]
20-49	99	570 566	1.74	14.4 (5.31-39.1)^e^	9.27 (3.39-25.4)^e^	10.3 (3.79-28.2)^e^
50-64	150	124 723	12.0	102.0 (37.8-275.1)^e^	14.7 (5.26-41.3)^e^	19.0 (6.81-52.9)^e^
≥65	210	70 164	29.9	257.3 (95.7-691.4)^e^	15.3 (5.44-43.3)^e^	20.1 (7.15-56.5)^e^
Sex						
Women	227	581 201	3.91	1 [Reference]	1 [Reference]	1 [Reference]
Men	236	516 752	4.57	1.18 (0.98-1.41)	NA	NA
Hypertension						
No	101	967 727	1.04	1 [Reference]	1 [Reference]	1 [Reference]
Yes	362	130 226	27.8	27.3 (21.9-34.1)^e^	2.27 (1.57-3.28)^e^	4.79 (3.52-6.51)^e^
Diabetes						
No	237	1 074 875	2.20	1 [Reference]	1 [Reference]	1 [Reference]
Yes	226	23 078	97.9	46.6 (38.8-56.0)^e^	6.44 (5.18-8.01)^e^	7.01 (5.63-8.73)^e^
Hyperlipidemia						
No	205	991 553	2.07	1 [Reference]	1 [Reference]	1 [Reference]
Yes	258	106 400	24.3	12.1 (10.1-14.5)^e^	1.07 (0.84-1.36)	1.24 (1.00-1.55)
Coronary artery disease						
No	271	1 027 461	2.64	1 [Reference]	1 [Reference]	1 [Reference]
Yes	192	70 492	27.2	10.6 (8.77-12.7)^e^	0.77 (0.62-0.95)^e^	0.82 (0.66-1.01)
Cerebrovascular disease						
No	418	1 089 131	3.84	1 [Reference]	1 [Reference]	1 [Reference]
Yes	45	8822	51.0	13.9 (10.2-19.0)^e^	1.18 (0.86-1.62)	1.25 (0.91-1.71)
Chronic kidney disease						
No	314	1 092 826	2.87	1 [Reference]	1 [Reference]	1 [Reference]
Yes	149	5126	290.7	109.7 (90.1-133.7)^e^	15.6 (12.5-19.4)^e^	16.6 (13.3-20.7)^e^
COPD						
No	348	1 035 830	3.36	1 [Reference]	1 [Reference]	1 [Reference]
Yes	115	62 123	18.5	5.73 (4.64-7.08)^e^	0.93 (0.74-1.17)	0.95 (0.75-1.19)
Chronic liver diseases						
No	361	1 009 792	3.57	1 [Reference]	1 [Reference]	1 [Reference]
Yes	102	88 161	11.6	3.33 (2.67-4.16)^e^	0.83 (0.66-1.06)	0.85 (0.67-1.08)
Systemic lupus erythematosus						
No	459	1 097 289	4.18	1 [Reference]	1 [Reference]	1 [Reference]
Yes	4	664	60.3	14.9 (5.58-40.0)^e^	6.90 (2.47-19.2)^e^	8.09 (2.88-22.7)^e^
Ankylosing spondylitis						
No	456	1 095 006	4.16	1 [Reference]	1 [Reference]	1 [Reference]
Yes	7	2947	23.8	5.95 (2.82-12.5)^e^	2.04 (0.96-4.33)	2.12 (1.00-4.50)
Rheumatoid arthritis						
No	461	1 096 966	4.20	1 [Reference]	1 [Reference]	1 [Reference]
Yes	2	987	20.3	4.97 (1.24-19.9)^f^	2.08 (0.51-8.39)	2.21 (0.55-8.96)
Sjögren syndrome						
No	462	1 097 579	4.21	1 [Reference]	NA	NA
Yes	1	374	26.7	6.61 (0.93-47.0)	NA	NA
Hepatitis C						
No	449	1 090 988	4.12	1 [Reference]	1 [Reference]	1 [Reference]
Yes	14	6965	20.1	5.10 (3.00-8.69)^e^	0.92 (0.52-1.63)	0.86 (0.48-1.52)
HIV						
No	462	1 097 354	4.21	1 [Reference]	NA	NA
Yes	1	599	16.7	4.07 (0.57-28.9)	NA	NA
Hepatitis B						
No	446	1 070 589	4.17	1 [Reference]	NA	NA
Yes	17	27 364	6.21	1.55 (0.95-2.51)	NA	NA
Statin use						
No	313	1 064 891	2.94	1 [Reference]	1 [Reference]	NA
Yes	150	33 062	45.4	16.9 (13.9-20.6)^e^	1.37 (1.08-1.73)^f^	NA
NSAID use						
No	106	539 822	1.96	1 [Reference]	1 [Reference]	NA
Yes	357	558 131	6.40	3.64 (2.92-4.54)^e^	1.07 (0.84-1.36)	NA
Antihypertensive medication use						
No	87	942 379	0.92	1 [Reference]	1 [Reference]	NA
Yes	376	155 574	24.2	27.5 (21.8-34.8)^e^	3.27 (2.23-4.78)^e^	NA

Abbreviations: COPD, chronic obstructive pulmonary disease; HPV, human papillomavirus; HR, hazard ratio; NA, not applicable; NSAID, nonsteroidal anti-inflammatory drug.

^a^ Incidence rate per 10 000 person-years.

^b^ Multivariable analysis including age, comorbidities (ie, hypertension, diabetes, hyperlipidemia, coronary artery disease, cerebrovascular disease, chronic kidney disease, COPD, chronic liver diseases, systemic lupus erythematosus, ankylosing spondylitis, rheumatoid arthritis, and hepatitis C), and use of statin, NSAID, or antihypertensive medications.

^c^ Multivariable analysis including age and comorbidities (ie, hypertension, diabetes, hyperlipidemia, coronary artery disease, cerebrovascular disease, chronic kidney disease, COPD, chronic liver diseases, systemic lupus erythematosus, ankylosing spondylitis, rheumatoid arthritis, and hepatitis C).

^d^ *P* < .01.

^e^ *P* < .001.

^f^ *P* < .05.

In age subgroup analysis, compared with the age-matched non-HPV subgroups, individuals aged 50 to 64 years with HPV infection had a significantly lower risk of developing ESKD (adjusted HR, 0.48; 95% CI 0.34-0.68; *P* < .001), while there was no significant association between HPV infection and ESKD risk for the other age subgroups. The interaction for the age subgroups was not significant. In the sex subgroup analysis, individuals of both sexes with HPV had a lower risk of developing ESKD (women: adjusted HR, 0.73; 95% CI, 0.56-0.95; *P* = .02; men: adjusted HR, 0.71; 95% CI, 0.55-0.92; *P* = .009). However, the interaction for the sex subgroups was not significant. In the comorbidities subgroup analysis, compared with matched individuals without HPV infection, those with HPV infection and any comorbidity had a decreased risk of subsequent ESKD development (adjusted HR, 0.71, 95% CI 0.58-0.86; *P* < .001). However, the interaction for the comorbidities subgroup was not significant. Among people with HBV infection, there was no significant association of HPV infection with ESKD risk (adjusted HR, 1.09; 95% CI, 0.38-3.14), but there was a significant reduction in ESKD risk for individuals with HPV infection and no HBV infection (adjusted HR, 0.72; 95% CI, 0.60-0.87; *P* < .001). However, the interaction was not significant by subgroup for HBV. Among people without HCV, there was a significant negative association of HPV infection with ESKD (adjusted HR, 0.71; 95% CI, 0.59-0.85; *P* < .001), but there was no reduction in ESKD risk for individuals with HPV infection and HCV infection. There was also a significant interaction by HCV subgroup (*P* for interaction = .04) (Table 3). The Kaplan-Meier curve revealed that the cumulative incidence of ESKD was decreased in the HPV group compared with the non-HPV group (log-rank *P* = .003) (Figure).

**Table 3.** Subgroup Analysis

Variables	Without HPV			With HPV			HR (95% CI)		
	Events, No.	Person-years	Rate^a^	Events, No.	Person-years	Rate^a^	Crude	Adjusted^b^	*P* value for interaction
Age, y									
<20	2	164 071	0.12	2	168 429	0.12	0.97 (0.14-6.88)	0.58 (0.07-5.09)	.75
20-49	56	284 299	1.97	43	296 266	1.50	0.76 (0.51-1.13)	0.80 (0.53-1.20)	
50-64	96	61 836	15.5	54	62 887	8.59	0.55 (0.40-0.77)^c^	0.48 (0.34-0.68)^c^	
≥65	107	33 377	32.1	103	36 786	28.0	0.86 (0.66-1.13)	0.90 (0.69-1.19)	
Sex									
Women	133	289 595	4.59	94	291 606	3.22	0.70 (0.54-0.92)^d^	0.73 (0.56-0.95)^e^	.43
Men	128	253 989	5.04	108	262 763	4.11	0.81 (0.63-1.05)	0.71 (0.55-0.92)^d^	
Comorbidity^f^									
No	27	412 227	0.65	20	413 263	0.48	0.73 (0.41-1.31)	0.77 (0.43-1.37)	.95
Yes	234	131 357	17.8	182	141 106	12.9	0.72 (0.60-0.88)^d^	0.71 (0.58-0.86)^c^	
Diabetes									
No	128	532 245	2.40	109	542 630	2.01	0.83 (0.65-1.08)	0.85 (0.66-1.11)	.23
Yes	133	11 339	117.3	93	11 738	79.2	0.67 (0.51-0.87)^d^	0.65 (0.50-0.85)^d^	
Hepatitis C									
No	257	540 042	4.76	192	550 946	3.48	0.73 (0.61-0.88)^d^	0.71 (0.59-0.85)^c^	.04
Yes	4	3542	11.3	10	3423	29.2	2.56 (0.80-8.15)	1.85 (0.54-6.39)	
Hepatitis B									
No	251	530 319	4.73	195	540 270	3.61	0.76 (0.63-0.92)^d^	0.72 (0.60-0.87)^c^	.77
Yes	10	13 265	7.54	7	14 099	4.97	0.66 (0.25-1.73)	1.09 (0.38-3.14)	
Chronic kidney disease									
No	184	541 047	3.40	130	551 780	2.36	0.69 (0.55-0.86)^d^	0.64 (0.51-0.80)^d^	.17
Yes	77	2537	30.5	72	2589	278.1	0.92 (0.66-1.26)	0.95 (0.68-1.32)	
NSAID use									
No	70	285 202	2.49	35	254 620	1.37	0.55 (0.36-0.82)^d^	0.53 (0.35-0.80)^d^	.16
Yes	190	258 381	7.35	167	299 749	5.57	0.75 (0.61-0.93)^d^	0.79 (0.64-0.97)^e^	

Abbreviations: HPV, human papillomavirus; HR, hazard ratio; NSAID, nonsteroidal anti-inflammatory drug.

^a^ Incidence rate per 10 000 person-years.

^b^ Multivariable analysis including age, comorbidities (ie, hypertension, diabetes, hyperlipidemia, coronary artery disease, cerebrovascular disease, chronic kidney disease, chronic obstructive pulmonary disease, chronic liver diseases, systemic lupus erythematosus, ankylosing spondylitis, rheumatoid arthritis, and hepatitis C), and use of statin, NSAID, or antihypertensive medications.

^c^ *P* < .001.

^d^ *P* < .01.

^e^ *P* < .05.

^f^ Individuals with any comorbidity (ie, hypertension, diabetes, hyperlipidemia, coronary artery disease, cerebrovascular disease, chronic kidney disease, chronic obstructive pulmonary disease, chronic liver diseases, systemic lupus erythematosus, ankylosing spondylitis, rheumatoid arthritis, Sjögren syndrome, hepatitis C, HIV, or hepatitis B) were classified into the comorbidity group.

**Figure.**
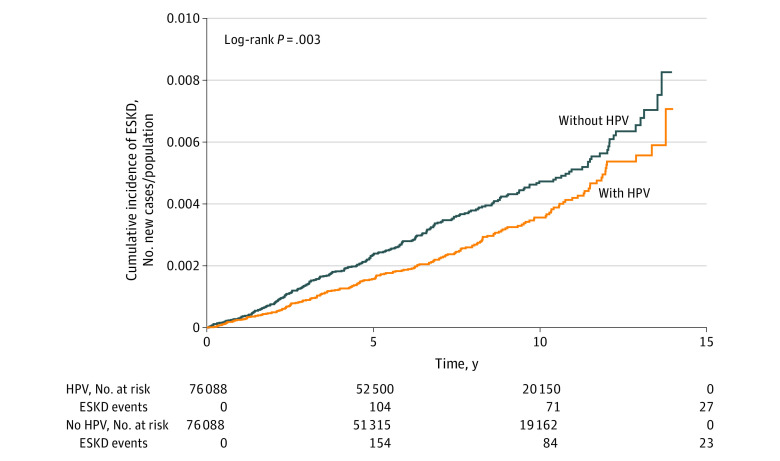
Cumulative Incidence of End-Stage Kidney Disease (ESKD) HPV indicates human papillomavirus.

## Discussion

In this retrospective cohort study using nationwide population-based data over 12 years, a prior diagnosis of HPV was associated with a 28% reduction of risk of developing ESKD. The age cluster phenomenon, with 80.0% of the patients in the HPV study group age 49 years and younger, may be due to the increased prevalence of HPV among younger age groups. Furthermore, stratified analysis revealed that HPV infection was associated with a decreased risk of ESKD in both sexes and for people aged 50 to 65 years. HPV infection may be an independent and distinct factor with a protective association with the development of ESKD even after adjusting for baseline characteristics, comorbidities, and comedications. However, an interaction between HCV status and HPV infection status associated with the development of ESKD was also observed in this study. In the presence of HCV, the decreased association between HPV and ESKD was not observed. The underlying mechanism to explain how HPV infection may decrease the risk of developing ESKD remains unclear. Further studies verifying the potential protective association between HPV infection and ESKD are warranted.

Additionally, the interaction of HCV with HPV and the subsequent risk of ESKD warrants further study. Our stratified analysis suggested that HCV may be a mediator between HPV infection and the risk of ESKD. HCV infection is a documented risk factor for CKD and progression to ESKD. Based on a 2018 review,^28^ HCV infection is associated with a higher incidence and faster progression of CKD, which may support our findings. Adequate anti-HCV therapy in patients with CKD improves long-term kidney and patient survival, so clinical suggestions of anti-HCV treatment are warranted.^29^ The interaction between HPV and HCV in oncogenesis was not investigated thoroughly in previous studies to our knowledge. We know that HPV and HCV involve p53 tumor suppressor pathway deregulation.^30^ In a 2016 case-control study of head and neck cancers,^31^ HCV was found to be associated with nonoropharyngeal and HPV-positive oropharyngeal head and neck cancers. That study by Mahale et al^31^ also suggested that the HPV oncoproteins E6 and E7 directly inhibit type 1 interferon signaling, which is crucial for HCV intracellular replication. Thus, HPV may facilitate the proliferation of HCV in oropharyngeal cells. Moreover, an oncogenic synergistic role in head and neck cancer development was suggested based on the induction of p53 degradation by HPV E6 protein and Rb degradation by the HCV NS5B protein.^31^ Although HPV and HCV seem to share similar oncogenic pathways, their interaction in kidney function remains unclear. Further investigations are still needed to elucidate the associations among HPV, HCV, and ESKD.

We identified a possible association between HPV infection and ESKD, although the underlying mechanism remains unclear. The unselected large sample size, long-term follow-up period (up to 12 years), and use of population-based data were advantages of using the national database. Several rigorous statistical models were applied in our study. Additionally, strict inspection of applications for certification helped ensure the accuracy of ESKD diagnosis acquired from the catastrophic diseases database. As is mandatory in Taiwan, ESKD diagnosis was confirmed by 2 nephrologists who reviewed the original medical records, laboratory data, and imaging findings.

### Limitations

This study had several limitations. First, our cohort study design was subject to biases regarding confounding adjustment. Despite using 2 statistical models to adjust for confounding factors, bias may have occurred. Second, risk factors, such as lifestyle and family history of ESKD, are not recorded in the NHIRD. To minimize bias, we applied proxy measures to adjust other associated, unrecorded information, such as *ICD-9-CM* codes for diabetes, hypertension, hyperlipidemia, COPD, coronary artery disease, and cardiovascular disease. Third, no reports on laboratory factors, such as serum creatinine level or estimated glomerular filtration rate, were available in NHIRD. However, a 2015 study^32^ mentioned the acceptable accuracy of CKD diagnostic codes using the standard CKD definition (estimated glomerular filtration rate <60 mL/min/1.73 m^2^) and a regional hospital data set for validation (positive predictive value, 90.4).

Fourth, the study may have been had a selection bias, and one of the major concerns was avoiding such bias. Presumably, many of the patients with HPV had been infected for a long period before detection. This limitation makes matching on the index date difficult. The look-back period before the index date was made sufficiently long, from 1995 to 2000, and HPV events of all age groups (from 0-100 years) were included. Inevitably, some patients with asymptomatic HPV infection do not submit claims for medical services, and thus, these patients may have been classified into the comparison group in this study. However, if HPV is associated causally with subsequent ESKD, patient misclassification would bias the estimated HRs toward the null.

## Conclusions

In this population-based cohort study, the presence of HPV infection was associated with a reduction of the risk of developing ESKD. An interaction between HCV status and HPV infection status associated with the development of ESKD was also observed in this study. Future detailed studies of the mechanisms involved may yield targets for interventions that could prevent or delay the development of ESKD.
